# The prognostic value of immune-nutritional status in metastatic colorectal cancer: Prognostic Nutritional Index (PNI)

**DOI:** 10.1007/s00520-024-08572-6

**Published:** 2024-05-23

**Authors:** Merve Keskinkilic, Huseyin Salih Semiz, Evrim Ataca, Tugba Yavuzsen

**Affiliations:** 1 Department of Medical Oncology, Faculty of Medicine, Dokuz Eylul University, Izmir, Turkey; 2 Department of Medical Oncology, Institute of Oncology, Dokuz Eylul University, Izmir, Turkey; 3 Deparment of Internal Medicine, Mus State Hospital, Mus, Turkey

**Keywords:** Colorectal cancer (CRC), Immune status, Metastasis, Nutritional assessment, Prognostic factors

## Abstract

**Backround and purpose:**

A low Prognostic Nutritional Index (PNI) value, which reflects immune nutrition and inflammation around the tumor, is associated with an unfavorable prognosis, and it was aimed to reveal its prognostic value in metastatic colorectal cancer (CRC).

**Methods:**

In our retrospective cross-sectional study, patients with a diagnosis of metastatic colorectal disease without active infection, between January 2010 and December 2016 were included. The PNI values at the time of diagnosis were calculated according to the formula (10 × serum albumin (g/dL)) + (0.005 × total lymphocyte value).

**Results:**

The mean PNI value of 253 patients included in the study was 46.6. While 53.75% (*n* = 136) of the patients had a PNI value of 46.6 and above, 46.25% (*n* = 117) had a PNI value below 46.6. The overall survival (OS) of the group with a PNI of 46.6 and above was statistically significantly longer (53.06 months vs 38.80 months, *p* = 0.039). The PFS duration of the group with PNI below 46.6 was 25.66 months, while the PFS duration of the group with PNI above 46.6 was not reached (*p* = 0.265).

**Conclusion:**

PNI is a simple and inexpensive index that evaluates the immunonutritional status, and it is a prognostic marker that can be easily used in patients with metastatic colorectal cancer as in other cancer types.

## Introduction

According to the 2020 GLOBOCAN data, colorectal cancer is the second most common cancer in women and the third most common cancer in men, and when evaluated in terms of cancer-related death, it is the third most common cancer that causes death in both genders [1]. According to SEER database data, approximately 22% of patients have distant metastases at the time of diagnosis, while the 5-year overall survival rate of metastatic patients is 15.1% [2]. Despite recent advances in multidisciplinary approaches and targeted therapies, and immunotherapy, including surgery, chemotherapy, and radiotherapy, the mortality rate of colorectal cancer remains high, especially in patients with distant metastases or relapses after treatment.

When we evaluate the prognostic factors in patients with metastatic colorectal cancer, whose mortality rate is still high, biological, genetic, molecular, and tumor tissue–related characteristics have an impact on prognosis. In addition to these known prognostic factors, many studies are seeking answers to these questions by searching for biomarkers that can predict mortality and relapse and have a prognostic value. While investigating these biomarkers, it has been shown that inflammation and immune response mechanisms in the tumor microenvironment play an important role in tumor progression, as in Hanahan’s hallmark of cancer definition [3]. After obtaining this information, many studies were initiated on inflammation markers that would reflect the status of the tumor. Recent studies show that in addition to these known prognostic factors, patient-related nutrition, inflammation, and immune status also affect prognosis. There are studies showing that inflammation, immunity, and nutritional status are effective in the prognosis of colorectal cancer. For this reason, the search for a simple, inexpensive, and applicable marker to predict prognosis in addition to existing prognostic factors is the subject of many studies.

C-reactive protein (CRP), albumin, neutrophil, lymphocyte, platelet, and combined formulations of these values include the neutrophil to lymphocyte ratio (NLR), lymphocyte to monocyte ratio (LMR), platelet to lymphocyte ratio (PLR), lymphocyte to C-reactive protein ratio (LCR), systemic inflammation score (SIS), and Prognostic Nutrition Index (PNI), and the prognostic value and importance of these markers have been tried to be understood through various studies, and studies on these markers in many types of cancer are still continuing at full speed [4–6]. The midpoints of all these markers can be calculated with a simple blood count parameter, are inexpensive, and reflect the patient’s immune and nutritional status, which are prognostic factors.

First, Onodera et al. [7] developed an inexpensive and simple PNI that evaluates the immune and inflammation status in nutritionally affected cancer patients, showing its prognostic value in many malignancies and malignancy-related surgeries. PNI, which was developed to evaluate immune and nutritional status, which is understood to be important in prognosis, is one of these markers. The PNI value is calculated with the formula (10 × albumin (g/dL) + 0.005 × total lymphocyte count (per mm^3^)) and reflects the serum albumin value in the formulation and the nutritional status, and the absolute lymphocyte value reflects the immunological status [7].

It has been shown that in some types of cancer, a low PNI value has a poor prognostic value and is associated with short overall survival time and poor postoperative outcomes [8–13].

While cancer-related malnutrition affects approximately 85% of cancer patients, malnutrition increases up to 30–60% in patients diagnosed with colorectal cancer among cancer types [14].

Therefore, we aimed to reveal the relationship between PNI and prognosis and overall survival in patients with metastatic colorectal cancer, considering that there is no sufficient and information available in the literature regarding the prognostic value of PNI in metastatic colorectal cancer and the thought that PNI value will have prognostic importance in patients with colorectal cancer whose nutritional status is highly affected.

## Material and methods

### Study population

In our retrospective cross-sectional study, patients with a diagnosis of metastatic colorectal disease, aged 18 years and older who were followed up in the Dokuz Eylul University, Faculty of Medicine, Department of Medical Oncology, between January 2010 and December 2016 were included. Patients were included on the basis of the following criteria: (1) the patient was diagnosed with biopsy stage IV colorectal cancer; (2) being Eastern Cooperative Oncology Group (ECOG) Performance Status 0-2; (3) the patient has been followed in the clinic for at least 3 months; (4) the patient has received any treatment (chemotherapy or tyrosine kinase inhibitor) for colorectal cancer; and (5) the PNI score can be calculated from laboratory parameters which blood samples were obtained within time of diagnosis. Patients were excluded according to the following criteria: (1) received anti-inflammatory medication; (2) patients with synchronous and metachronous tumors; (3) patients with serious complications or acute and chronic inflammatory diseases of any type; (4) patients with inflammatory and autoimmune diseases; (5) patients who received blood product transfusion within 1 month before cancer treatment; (6) patients who are using enteral nutrition solution; (7) patients who were untreated for serious cardiovascular disease (stage 3 or 4 according to the New York Heart Association classification). Sociodemographic and clinicopathological data, treatment-related characteristics of the patients, and laboratory parameters of the patients were obtained retrospectively from the hospital database. In order to evaluate the presence of clinical metastases, computerized tomography and F-18 fluorodeoxyglucose (FDG)-positron emission tomography/computed tomography (PET/CT) results in the hospital database were scanned.

### Prognostic Nutritional Index (PNI)

The PNI was calculated as follows: (10 × serum albumin (g/dL)) + (0.005 × total lymphocyte value) [7]. The dependent variable of the study was the Prognostic Nutritional Index calculated by albumin and lymphocyte values ontime or within 1 week time of diagnosis. The optimal cut-off value before treatment was taken as 46.6 for the PNI score, and the patients were divided accordingly into two groups as low PNI (< 46.6) and high PNI (≥ 46.6).

#### Follow-up

Routine follow-up of all included patients was performed every 3 months. Follow-up examinations include routine laboratory examinations (in addition CEA, CA 19–9), computed tomography (CT), magnetic resonance imaging (MRI), or PET/CT when necessary.

Survival information and outcomes were obtained from clinical records during follow-up. The end of follow-up is until the patient dies or is lost to follow-up or until March 2022.

Patients with a life expectancy of 0 months were excluded from the study. Overall survival (OS) was defined as the time from the date of diagnosis to death or last follow-up. Progression-free survival (PFS) was defined as the time from the date of diagnosis until progression, relapse, death, or last follow-up, whichever came first.

### Treatment response and toxicity assessment

Tumor staging was performed according to the eighth edition of the American Joint Committee on Cancer (AJCC) and the Union for International Cancer Control (UICC) TNM stage classification [15]. Response assessments were performed according to the Response Evaluation Criteria in Solid Tumors (RECIST) v1.1 guidelines [16]. Toxicity assessments were based on the National Cancer Institute Common Toxicity Criteria (NCI-CTC) [17].

### Statistics analyses

Since our study was a retrospective, cross-sectional study, sample size was not calculated because all patients who met the inclusion criteria were included in the study. Descriptive statistics (mean ± standard deviation, percentage (%)) was used as appropriate for statistical analysis. The conformity of the data to the normal distribution was evaluated by Kolmogorov–Smirnov and Shapiro–Wilk tests. Data suitable for normal distribution were reported with mean and standard deviation (SD), while data not suitable for normal distribution were reported with median and interquartile range (IQR). In addition to descriptive statistics, chi-square and Fisher’s exact tests were used for categorical variables in the evaluation of the data. The Mann–Whitney *U* test and the Wilcoxon signed ranks test were used to compare the variables indicated by the measurement. Receiver operating characteristic (ROC) curve analysis was applied to select the most appropriate cut-off point for PNI to discriminate patients at high risk of cancer-related death. However, since a value with sufficient sensitivity and specificity could not be obtained from the ROC curve, the mean value of PNI in the tumor group was determined as the cut-off point. The patients were divided into two groups as those with a mean PNI value and above and those with a mean value below the mean and compared in terms of overall survival and progression-free survival. To evaluate the effect of PNI on survival, univariate and multivariate Cox regression models were applied to identify the best predictive variables. The Kaplan–Meier method was used to estimate PFS and OS while the log-rank test was used to investigate the difference in survival median follow-up time in the study that was calculated using reverse Kaplan–Meier. All data were analyzed using the IBM SPSS (Statistical Package for the Social Sciences, version 24.0) package program. The *p* value was used to determine statistical significance in all tests performed, and *p* < 0.05 was considered statistically significant.

### Ethics committee approval

This study was performed in line with the principles of the Declaration of Helsinki. Approval was granted by the Non-Invasive Research Ethics Committee of Dokuz Eylul University (Date: 13.04.2022/Number: 2022/14–14). Written informed consent form was obtained from all patients included in the study.

## Results

### Patient characteristics

The median age of 253 patients included in the study was 61.0 (range, 27.1–88.6) years, and 61.7% (*n* = 156) were male. Fifty-five point three percent (*n* = 140) of the patients were ECOG PS-0, 31.6% (*n* = 80) were ECOG PS-1, and 13.1% (*n* = 33) were ECOG PS-2. The most common comorbid disease in patients is hypertension with 39.5% (*n* = 100) and this is followed by diabetes with 23.3% (*n* = 59), coronary artery disease with 14.2% (*n* = 36), and chronic obstructive pulmonary disease with 7.9% (*n* = 20), respectively. While 47.03% (*n* = 119) of the patients included in the study did not have any comorbidity, 27.2% (*n* = 69) had only one comorbidity and 25.6% (*n* = 65) had two or more comorbidities.

### Clinicopathological and treatment characteristics

The majority of the population of 253 patients was 88.1% (*n* = 223) diagnosed with colon cancer, and the remainder of the population was 11.9% (*n* = 33) diagnosed with rectal cancer. When we evaluated the patients according to primary tumor location, the majority of the population consisted of the left-sided colon in 74.3% (*n* = 188) and the right-sided in 25.7% (*n* = 65) of the population. The most common site of metastasis was the liver with a rate of 81% (*n* = 205), followed by lung metastases at a rate of 60.5% (*n* = 153) and lymph node metastases at 54.2% (*n* = 137). Clinicopathological characteristics of the study population are shown in Table 1.

**Table 1:** Clinicopathological characteristics of the study population

Characteristic	% (*n*)
Diagnosis	
Colon	88.1% (*n* = 223)
Rectum	11.9% (*n* = 30)
Primary tumor site	
Left colon	74.3% (*n* = 188)
Right colon	25.7% (*n* = 65)
Metastasis sites	
Liver	81% (*n* = 205)
Lung	60.5% (*n* = 153)
Lymph node	54.2% (*n* = 137)
Peritoneum	51.8% (*n* = 131)
Bone	13.0% (*n* = 33)
Other (adrenal, ovarian, cranial, bladder)	21.7% (*n* = 55)
RAS status	
Mutant	51.8% (*n* = 131)
Wild	48.2% (*n* = 122)
BRAF status	
Wild	95.7% (*n* = 242)
Mutant	4.4% (*n* = 11)

The most preferred chemotherapy protocol as first-line treatment in the metastatic stage was FOLFOX4 with a rate of 68% (*n* = 172), followed by FOLFIRI with a rate of 14.2% (*n* = 36) and XELOX with a rate of 9.9% (*n* = 25). Fifty-eight point six percent (*n* = 131) of the study population were KRAS (codons 12, 13, and 61) mutant, 4.7% were NRAS (codons 12, 13, and 61) mutant, and 4.4% were BRAF mutant, and the rate of use of anti-VEGF-targeted biological drugs (bevacizumab) as first-line therapy in combination with chemotherapy was 71.9% (*n* = 182) and the rate of use of anti-EGFR targeted biological agents (cetuximab and panitimumab) is 26.9% (*n* = 68). Patients received mean 2.16 (std ± 0.74) line of systemic chemotherapy in the metastatic stage. While 79.0% (*n* = 200) of the patients were able to receive second-line treatment, 37.5% of them could receive third-line treatment. While 41.9% (*n* = 106) of the patients had metastasis surgery at the metastatic stage, 22.5% (*n* = 57) received palliative radiotherapy. Treatment-related features are shown in Table 2.

**Table 2:** Treatment-related characteristics of the study population

Characteristic	% (*n*)
First-line treatment protocol	
FOLFOX4	68% (*n* = 172)
FOLFIRI	14.2% (*n* = 36)
XELOX	9.9% (*n* = 25)
Other (capecitabine, Tomox, FOLFOX6)	7.9% (*n* = 20)
First-line anti-VEGF treatment	
Bevacizumab	71.9% (*n* = 182)
Did not use	28.1% (*n* = 71)
First-line anti-EGFR treatment	
Did not use	73.1% (*n* = 185)
Cetuximab	22.1% (*n* = 56)
Panitumumab	4.7% (*n* = 12)
Second-line treatment protocol	
FOLFIRI	70% (*n* = 140)
FOLFOX4	15.5% (*n* = 31)
XELOX	2% (*n* = 4)
Other (capecitabine, Tomox, MDG)	12.5% (*n* = 25)
Tyrosine kinase inhibitor treatment (regorafenib)	
Received	21.3% (*n* = 54)
Not received	78.7% (*n* = 199)

### Treatment response evaluation and survival analysis

Using the last seen date as the data cut-off point, follow-up period in our study was 28.1 months. In the entire study population, the disease control rate (DCR) at the 3rd month of the first-line treatment recipients was 91.7% (*n* = 232), while at the 6th month, the DCR was lower and was 61.3% (*n* = 155).

**Fig. 1:**
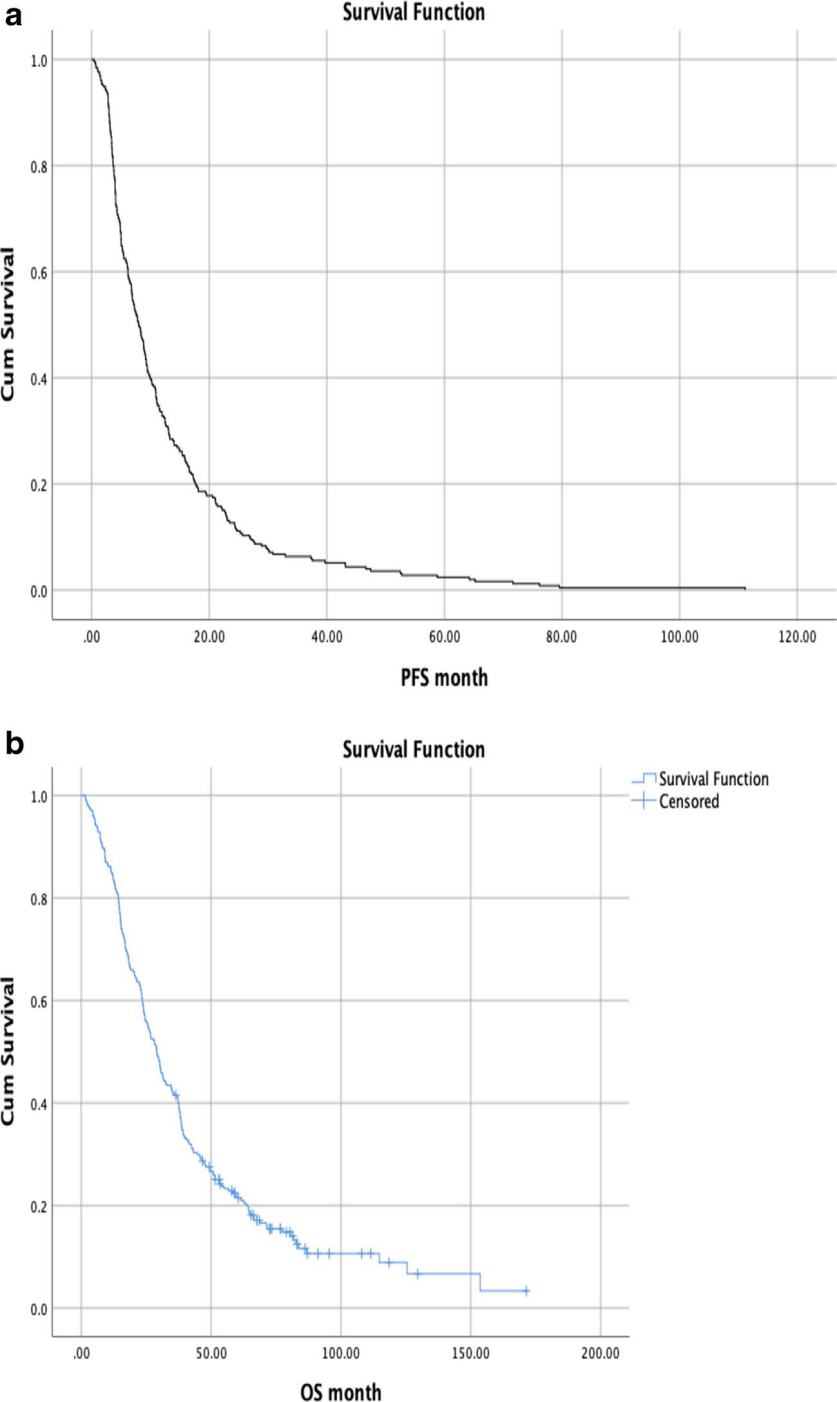
**a**, **b** Progression-free survival (PFS) and overall survival (OS) (all group)

In our study, using the last seen date as the data cut-off point, 13.8% of the patients were still alive, while median OS was 29.1 months (95% CI, 25.6–32.6) and median PFS was 12.7 months (95% CI, 10.9–14.5) at all of the study population (Fig. 1a, b). Tumors located in the left colon had a numerically longer median OS, although not statistically significant, when compared with tumors located in the right colon (26.0 months (95% CI, 22.8–29.2) vs 21.4 months (95% CI, 17.8–24.9), *p* = 0.195, respectively).

### Effect of Prognostic Nutritional Index on treatment response and survival

The mean PNI value of all the study groups was 46.6. While 53.7% (*n* = 136) of the patients had a PNI value of 46.6 and above, 46.2% (*n* = 117) had a PNI value below 46.6. In the group with PNI ≥ 46.6, the DCR was numerically higher, albeit not statistically, compared to the group with PNI < 46.6, and it was 56.8% vs 43.2% (*p* = 0.140).

**Fig. 2:**
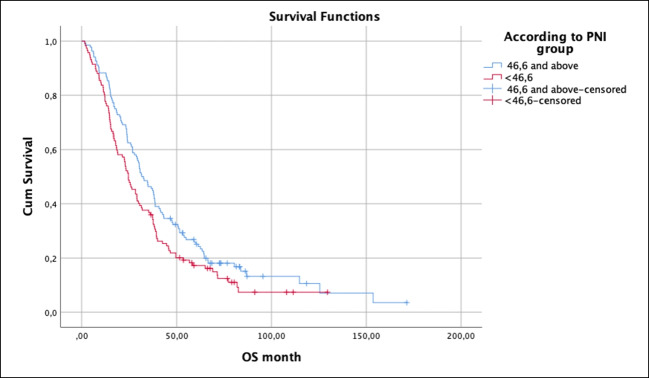
Overall survival according to the PNI group

Overall survival of the group with a PNI of 46.6 and above was statistically significantly longer (95% CI, 53.0 months vs 38.8 months, *p* = 0.039) (Fig. 2). In addition, the PFS duration of the group with PNI below 46.6 was 25.6 months, while the PFS duration of the group with PNI and above was not reached (*p* = 0.265).

**Fig. 3:**
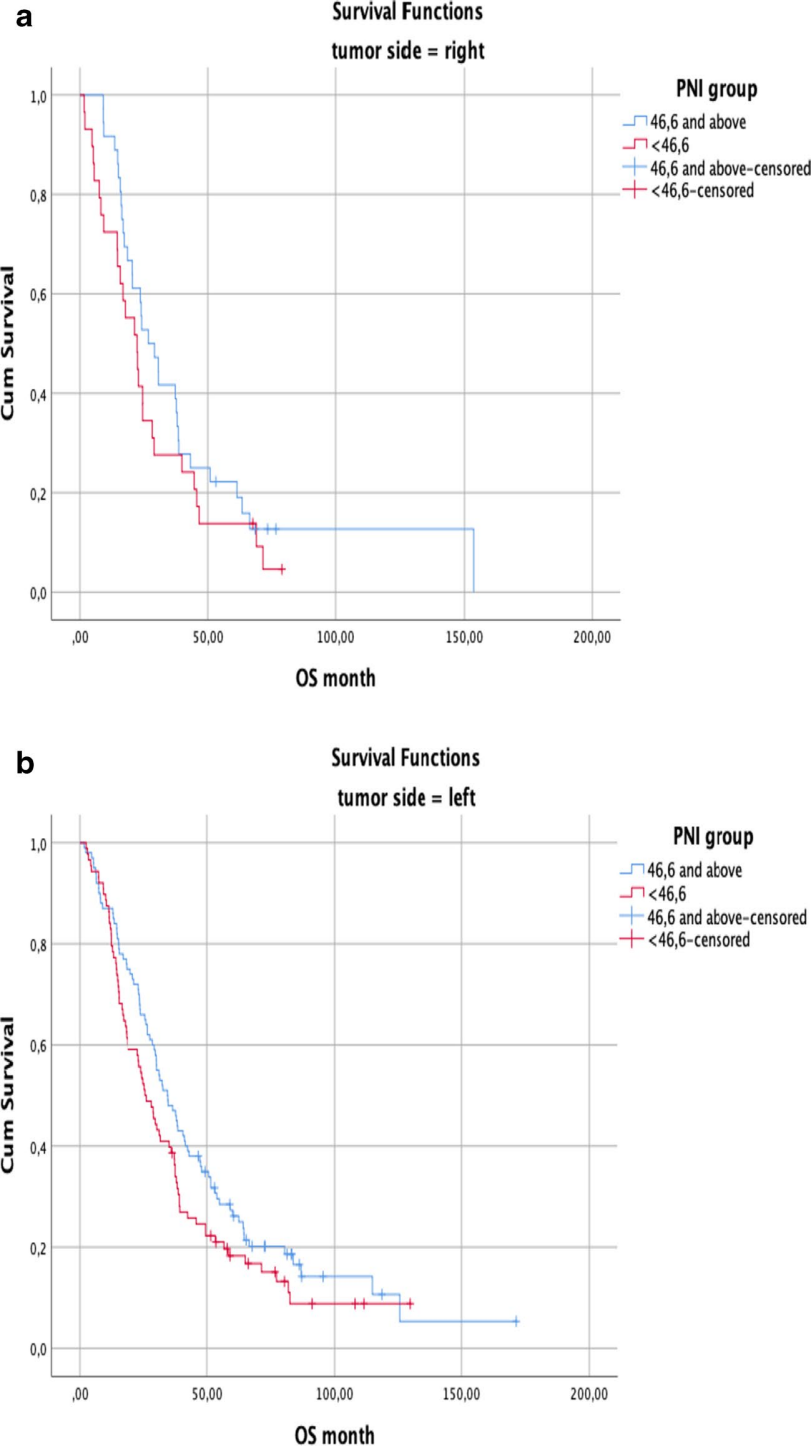
**a**, **b** According to tumor site overall survival (OS) in the PNI group

The group with PNI ≥ 46.6 in both the right and left colon had a statistically significantly longer OS (95% CI, 44.6 vs 27.2, 95% CI, 49.0 vs 38.2, *p* = 0.038), more prominent in right colon tumors (Fig. 3a, b).

**Fig. 4:**
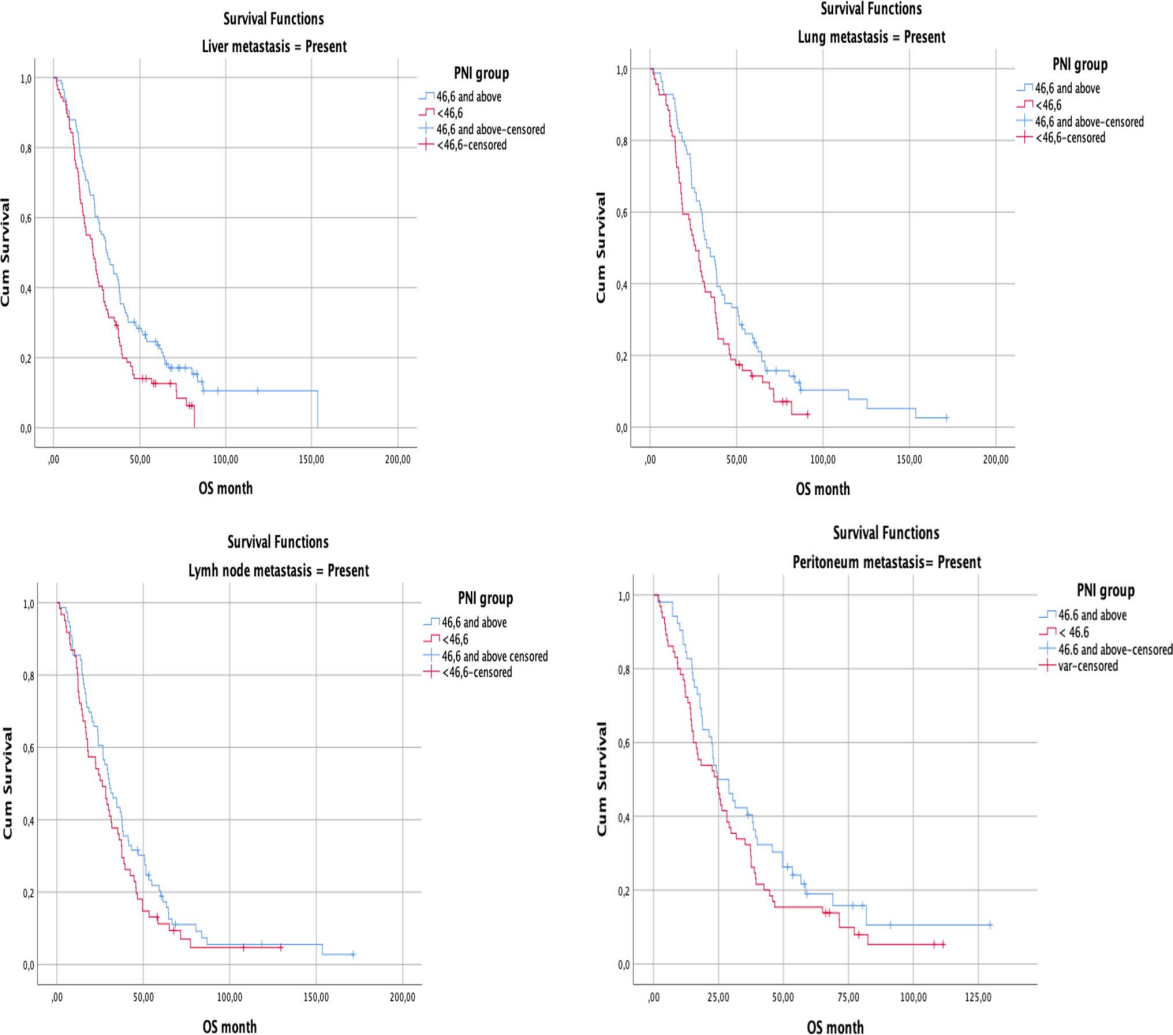
According to metastasis site overall survival in the PNI group

When evaluated according to metastasis sites, it was observed that the OS was significantly longer in the group with PNI 46.6 and above than in those with liver, lung, and lymph node metastases (*p* = 0.008, *p* = 0.021, *p* = 0.024, respectively). However, no statistically significant effect of PNI on OS could be demonstrated ın patients with peritoneal metastases (Fig. 4).

### Relationship between Prognostic Nutritional Index and other clinicopathological features

When the relationship between PNI and other clinicopathological features is evaluated, there was no relationship between PNI and gender, comorbidity status, tumor localization, and RAS mutation status (Table 3).

**Table 3:** Relationship between PNI and patient-related factors

Characteristic	PNI high (≥ 46.6) % (*n*) (*n* = 136)	PNI low (< 46.6) % (*n*) (*n* = 117)	*p* value
Sex			0.064
Male	66.9% (*n* = 91)	55.6% (*n* = 66)	
Female	33.1% (*n* = 45)	44.4% (*n* = 52)	
Comorbidity status			0.821
None	48.5% (*n* = 66)	45.3% (*n* = 53)	
Only one	25.7% (*n* = 35)	29.1% (*n* = 34)	
> 2 or more	25.7% (*n* = 35)	25.6% (*n* = 30)	
Tumor localization			0.733
Colon	87.5% (*n* = 119)	88.9% (*n* = 104)	
Rectum	12.5% (*n* = 17)	11.1% (*n* = 13)	
Kras status			0.690
Mutant	52.9% (*n* = 72)	50.4% (*n* = 59)	
Wild	47.1% (*n* = 64)	49.6% (*n* = 58)	

When the relationship between CEA, one of the tumor markers, and PNI was evaluated, the median CEA value was 1305.40 ng/mL in the group with a PNI value of PNI < 46.6, while the median CEA value was determined as 481.73 ng/mL in the group with PNI 46.6 and above. However, this result was not statistically significant (*p* = 0.294). When the relationship between CA 19–9 and PNI is examined, the median CA 19–9 value is 5268.37 in the group with a PNI value below 46.6, while the median CA 19–9 value is 1340.15, in the group with PNI value of 46.6 and above, was lower, although it did not reach statistical significance (*p* = 0.128).

Forty point three percent (*n* = 102) of the population consisted of patients aged 65 and over. The PNI values of 63.6% (*n* = 96) of those under 65 years of age and 39.2% (*n* = 40) of those aged 65 and older were 46.6 and above, and this difference was statistically significant (*p* =  < 0.001). In additon, the population aged 70 and over comprised 14.7% (*n* = 20) of the group with PNI 46.6 and above, and 35% (*n* = 41) of the group with PNI 46.6 below (*p* =  < 0.001). So, the younger population had a statistically significantly better immunonutritional status. The rate of those with good immunonutrition status in those who underwent metastasectomy was statistically significantly higher (63.2% vs 46.9%, *p* = 0.011).

## Discussion

Recent studies have shown that not only clinicopathological features but also immune and nutritional status are effective in predicting the prognosis and treatment of cancer [18]. Various indices have been developed to evaluate the immunonutritional status, and the PNI was created by using the absolute lymphocyte value reflecting the immune status and the serum albumin value reflecting the nutritional status [7, 18, 19]. The prognostic and predictive values of PNI have been investigated in various types of cancer [7, 20–22], and in this study, the prognostic value of PNI in patients diagnosed with metastatic colorectal cancer was evaluated and it was observed that the overall survival was significantly longer in the group with a higher PNI value consistent with the literature. In addition, the rate of disease control was higher in the group with high PNI, although it was not statistically significant, and the median PFS could not be reached in this group.

After it was understood that the immune and nutritional status had an effect on cancer prognosis, various parameters and indices reflecting the immune status (CRP, lymphocyte, albumin, neutrophil, NLR, PLR, MLR, pan-immune ınflammatıon value (PIV)) have been the subject of studies [23]. When these studies are evaluated, there are studies showing that inflammation, immunity, and nutritional status are also effective in the prognosis of colorectal cancer [23]. In addition to these inflammatory biomarkers, the PNI, a simple and inexpensive immunonutritional index developed by Onodera et al., is a marker that evaluates the nutritional status with the albumin in its formulation and the immune status with the absolute lymphocyte count, and has been shown to be an independent prognostic factor in many cancer types [7–9, 11–13, 20–22]. Its prognostic and predictive value in colorectal cancer, as in other types of cancer, has been investigated in various studies and its effect has been shown [18, 23–26].

In our study, we showed that the group with a high PNI value in patients with a diagnosis of metastatic colorectal cancer had a statistically significantly longer OS and PFS than the group with a low PNI value. When examining the literature, no relationship was found between PNI and death in a study by Zhou et al. [27], which included patients with locally advanced colorectal cancer, while a study by Uçar et al. [25] showed that high PNI values were associated with longer OS in metastatic CRC patients. Again, as a different design, Ikeya et al. calculated both pre-treatment and post-treatment PNI values of 80 patients with unresectable colorectal cancer and stated that those with higher PNI values both before and after treatment had a better prognosis [26]. Unlike other studies, Li et al. investigated the predictive effect of albumin to globulin ratio (AGR) along with PNI on the prognosis of colorectal cancer and stated that lower AGR and PNI values were associated with shorter OS and PFS [23].

In the Ikeya et al. study, no relationship was found between tumor location [26], tumor histology, CEA value, and PNI, except for gender. In our study, in addition to gender, there was no relationship between comorbidity, CEA, CA 19–9 tumor location, KRAS status, and PNI.

In both studies conducted by Ikeya et al. and Uçar et al. in patients with metastatic CRC, the relationship of metastasis sites and metastasectomy with PNI was not evaluated [25, 26], but in our study, it was observed that a higher PNI value was a better prognostic factor in patients with liver, lung, and lymph node metastases and those who underwent metastasectomy.

Although PNI was not evaluated in the geriatric colorectal cancer group when the literature was examined, PNI, which is an indicator of immune nutritional status, was found to be higher in the group under 65 years of age. In the geriatric population, the Geriatric Nutrition Risk Index (GNRI) calculated by using body weight and albumin instead of PNI was established, and survival and prognosis were found to be better in those with higher levels [28].

When we assessed at the previously published studies, the threshold value of PNI was between 45 and 57, and the PNI value was 46.6 in our study, which was found to be consistent with the literatüre [29].

In this study, it was revealed that PNI, which is a marker of immune nutritional status, has prognostic value in addition to known biological, genetic, and tumor pathology–related prognostic factors in patients with metastatic colorectal cancer, where nutritional status is affected quite frequently and the immune status is important. Such that, it was concluded that higher PNI values are associated with longer overall survival, but PFS is still not reached. It was revealed that the PNI value was statistically significantly higher, especially in the young population and those who could undergo metastasectomy.

Our study has some limitations. Among these, the fact that it is retrospective, it is a heterogeneous group, and the PNI value has different cut-off values, as in the studies, can be counted. More comprehensive prospective studies to determine that PNI is an independent prognostic factor will contribute to its prognostic value to determine a definitive PNI cut-off. This study will help to further elucidate the role of PNI in this patient group.

## Conclusion

The Prognostic Nutritional Index (PNI) is a simple, inexpensive, and applicable prognostic index in patients with metastatic colorectal cancer, and it is one of the new-generation prognostic factors that reflect individual patient-related immune status as well as classical TNM and genetic prognostic factors.

## Data Availability

No datasets were generated or analysed during the current study.

## Abbreviations

*AJCC*: Eighth Edition of the American Joint Committee on Cancer
*AGR*: Albumin to globulin ratio
*CRC*: Colorectal cancer
*ECOG-PS*: Eastern Cooperative Oncology Group—Performance Status
*FDG*: F-18 Fluorodeoxyglucose
*GNRI*: Geriatric Nutrition Risk Index
*LMR*: Lymphocyte to monocyte ratio
*LCR*: Lymphocyte to C-reactive protein ratio
*NCI-CTC*: National Cancer Institute Common Toxicity Criteria
*NLR*: Neutrophil to lymphocyte ratio
*OS*: Overall survival
*PET/CT*: Positron emission tomography/computed tomography
*PIV*: Pan-immune inflammation value
*PLR*: Platelet to lymphocyte ratio
*PNI*: Prognostic Nutritional Index
*PFS*: Progression-free survival
*RECIST*: Response Evaluation Criteria in Solid Tumors
*ROC*: Receiver operating characteristic
*SIS*: Systemic Inflammation Score
*UICC*: Union for International Cancer Control
